# Associations of phase angle with platelet-activating factor metabolism and related dietary factors in healthy volunteers

**DOI:** 10.3389/fnut.2023.1237086

**Published:** 2023-11-03

**Authors:** Paraskevi Detopoulou, Elizabeth Fragopoulou, Tzortzis Nomikos, Smaragdi Antonopoulou

**Affiliations:** Department of Nutrition and Dietetics, School of Health Science and Education. Harokopio University, Athens, Greece

**Keywords:** phase angle, bioelectrical impedance analysis, erythrocyte fatty acids, platelet-activating factor, dietary antioxidant capacity, Mediterranean diet

## Abstract

**Introduction:**

Phase angle (PA) is derived from bioelectrical impedance analysis (BIA). It reflects cell membrane function and decreases in disease. It is affected by inflammation, oxidative stress, and diet. Platelet-activating factor (PAF) is a potent inflammatory lipid mediator. Its levels, along with the activity of its metabolic enzymes, including CDP-choline:1-alkyl-2-acetyl-*sn*-glycerol-cholinephosphotransferase, acetyl-CoA:lyso-PAF-acetyltransferases, and PAF-AH/Lp-PLA_2_ are also related to dietary factors, such as the dietary antioxidant capacity (DAC). The aim of the study was to estimate whether the PAF metabolic circuit and related dietary factors are associated with PA in healthy volunteers.

**Methods:**

In healthy subjects, PAF, its metabolic enzyme activity, and erythrocyte fatty acids were measured, while desaturases were estimated. Food-frequency questionnaires and recalls were used, and food groups, macronutrient intake, MedDietScore, and DAC were assessed. Lifestyle and biochemical variables were collected. DXA and BIA measurements were performed.

**Results:**

Lp-PLA_2_ activity was positively associated with PA (rho = 0.651, *p* < 0.001, total population; rho = 0.780, *p* < 0.001, women), while PAF levels were negatively associated with PA only in men (partial rho = −0.627, *p* = 0.012) and inversely related to DAC. Estimated desaturase 6 was inversely associated with PA (rho = −0.404, *p* = 0.01, total sample). Moreover, the DAC correlated positively with PA (rho = 0.513, *p* = 0.03, women). All correlations were adjusted for age, body mass index, and sex (if applicable).

**Conclusion:**

PA is associated with PAF levels and Lp-PLA_2_ activity in a gender-dependent fashion, indicating the involvement of PAF in cell membrane impairment. The relationship of PA with DAC suggests a protective effect of antioxidants on cellular health, considering that antioxidants may inhibit PAF generation.

## Introduction

Bioelectrical impedance analysis (BIA) is a simple technique to assess body composition (1). Phase angle (PA) is calculated from the reactance (Xc) and resistance (R) ratios (BIA raw parameters) (1). Although its biological meaning in health and disease is not completely understood, PA is considered a measure of cell membrane function (1, 2). High values suggest intact or healthy cell membranes, while low values suggest cell death and impaired cell integrity/permeability and are documented in various diseases, such as malnutrition and cancer (1). Although cell membrane function and integrity can be altered by fatty acids (3), choline-based phospholipids (4, 5), and other dietary constituents, such as polyphenols (6) and antioxidants (7), scarce data exist on the relationship of the above constituents with PA. In addition, PA has been negatively related to inflammatory indices (such as C-reactive protein), cytokines (such as interleukin-6) (1, 8), and markers of oxidative stress (9, 10), and it has been positively related to the Mediterranean diet (11), to a pattern rich in animal proteins and potatoes in cancer patients (12), as well as to erythrocyte n-3 fatty acids (13, 14).

Platelet-activating factor (PAF) is an ether-linked glyceryl-phospholipid containing choline with an acetyl group in the *sn*-2 position that is responsible for its characteristic biological action. PAF was initially implicated in immediate hypersensitivity-type allergic reactions and platelet activation, while now it is considered the most potent inflammatory lipid mediator orchestrating inflammation, thrombosis, and disease pathophysiology (15–20). PAF is produced by various cells, among which endothelial cells, platelets, macrophages, monocytes, neutrophils, and mast cells are unique tissue immune cells that can be activated by numerous triggers. PAF also functions as an immunoregulatory mediator (21, 22) by modulating T- and B-cell activation and proliferation (23–25). It acts by binding to its specific receptor, namely PAFR, expressed by many cells and tissues (20), including mast cells, granulocytes, B-lymphocytes, dendritic cells, and macrophages (20, 21). The binding of PAF to its receptor stimulates complex cell signaling through the activation of G-proteins (19). Moreover, components of the bacterial wall, such as lipoteichoic acid and lipopolysaccharides, bind to PAFR, while the PAF-PAFR complex activates toll-like receptor 4 (TLR4), all supporting the idea that PAFR acts as an alternative system for innate immunity (26, 27). PAF is biosynthesized by the *de novo* and the remodeling pathway (20), key enzymes of which are CDP-choline: 1-alkyl-2-acetyl-*sn*-glycerol cholinephosphotransferase (PAF-CPT) and acetyl-CoA: lyso-PAF acetyltransferases (Lyso-PAF-AT), respectively (20). The remodeling pathway starts with the action of cytoplasmic phospholipase A_2_ (cPLA_2_) on the existing membrane ether-linked choline-containing phospholipids, resulting in the formation of lyso-PAF and the release of the fatty acids in the *sn*-2 glyceryl backbone position. The catabolism of PAF is mainly attributed to PAF acetylhydrolases (PAF-AH), which hydrolyze PAF, yielding lyso-PAF (20). The circulating isoform of PAF-AH is also known as lipoprotein-associated phospholipase A_2_ (Lp-PLA_2_) due to its binding to lipoproteins (28).

Studies with model membranes support the idea that ether phospholipid analogs, including PAF, can be inserted into membranes, but they may also perturb and disorder membrane bilayers at high concentrations (5). In addition, 1-O-octadecyl-2-O-methyl-*sn*-glycero-3-phosphocholine, a synthetic anti-tumor ether analog of PAF, has been reported to affect the properties of lipid rafts (29). PAF is related to membrane integrity (and possibly PA) either directly as a lipophilic membrane lipid constituent (5, 30, 31) or indirectly by modifying the antioxidant/inflammatory cellular milieu or the produced fatty acids (as a result of its metabolism) (20). For example, damage to cellular membranes results in the generation of PAF and PAF agonists, all binding to PAFR (32). In addition, PAFR stimulation induces the production of microvesicle particles (MVP), which are small membrane-bound particles, by activating the translocation of acid sphingomyelinase (aSMase) to the plasma membrane. PAFR stimulation activates the translocation of aSMase to the plasma membrane. This results in the hydrolysis of sphingomyelin (SM) and elevated ceramide production, both altering membrane integrity and fluidity (33–35).

Moreover, PAF and its metabolic enzymes are related to oxidative stress, inflammation, and dietary factors, all of which may affect PA (8–11, 13, 14). More specifically, oxidative stress activates Lyso-PAF-AT, leading to increased PAF levels (36). PAF can also be formed non-enzymatically during the oxidative modification of LDL, which reduces the activity of Lp-PLA_2_ (37). Additionally, PAF has been implicated in the production of reactive oxygen species (ROS) in neutrophils, eosinophils, endothelial cells, and monocytes (38–41). Recent data suggest that a healthy diet rich in antioxidants is associated with improved levels of PAF and Lp-PLA_2_ (42, 43), modulating PAF's pro-inflammatory actions and its metabolism (42). According to a systematic review of our group, several components of the Mediterranean diet, such as cereals, legumes, plant foods, fish, and wine, may modulate PAF actions and/or the activity of its enzymes in humans (44). PAF and its metabolic enzymes have also been associated with erythrocyte fatty acids (45).

To the best of our knowledge, there is no data connecting PAF levels or its metabolism with PA, while an association of platelet count with PA has been reported in patients with dengue fever (46). It can be hypothesized that PAF levels may affect cellular health and thus PA. Given the prognostic role of PA in diseases (47–51) where PAF is implicated (16, 52–54), the identification of potential modulators of PA is crucial. Thus, the aim of the present work was to study the associations of PA with PAF, its metabolic enzymes, and related dietary parameters in healthy participants.

## Materials and methods

### Subjects

The present study is a sub-analysis of a previous study investigating PAF and its enzymes in 106 participants (55). In the present analysis, all subjects from the above population with available data on BIA were considered (n = 39, 19 women). All participants gave written consent to participate. The protocol was approved by the Bioethics Committee of the university. The study was in accordance with the Declaration of Helsinki (1989) of the World Medical Association, as revised in 2013.

### Anthropometry, body composition, and PA

Weight, height, waist, and hip circumferences were measured as previously described (55). Body composition was assessed by DXA (Lunar Corporation, Brussels, Belgium) following the manufacturer's instructions. For each subject, the standard body composition analysis was performed (software version 4.6), and a region of interest (ROI) was manually defined as a quadrilateral box around the L1–L4 area, as previously described (55).

Whole-body BIA was performed with BODYSTAT (BODYSTAT, 1500) at a frequency of 50 kHz. After an overnight fast, the volunteers lied down on a bed with their arms and legs extended apart. The skin of hands and feet was disinfected with alcohol (70%) using disposable tissue paper. The electrodes were placed on the right hand (between the knuckles of the wrist) and on the right foot (on the ankle between the knuckles of the ankle). The subjects were at least 9–12 h without food (since they were in a fasted state to facilitate biochemical measurements) and 3 h without water. Regarding the menstrual cycle, women were neither in the middle of menses nor ±3 days, so as to avoid edemas.

The minimal distance between the electrodes was 5 cm. A single measurement was taken. PA was calculated from the Xc and R ratios as follows:


$$\begin{array}{l} PA=\left(Xc/R\right)\times \;180/\pi \end{array}$$

### Dietary assessment and lifestyle variables assessment

Two non-consecutive multiple-pass 24-h recalls were collected and assessed with the Nutritionist Pro™ software (Axxya Systems, Stafford, TX) expanded with local foods (56). Moreover, a semi-quantitative food frequency questionnaire (FFQ) was used (42) and Mediterranean diet adherence was assessed with the MedDietScore (57). The estimation of dietary antioxidant capacity (DAC) was based on previously published databases for raw Italian foods (58, 59), in which three different antioxidant assays were considered: the total radical-trapping antioxidant parameters (TRAP) (60), the ferric-reducing antioxidant power (FRAP) (61), and the Trolox-equivalent antioxidant capacity (TEAC) (62). More particularly, in the present study, 14 fruits, 17 vegetables, five types of legumes, six beverages, 17 composite foods, chocolate, honey, marmalade, nuts, and four cereal-based products were assigned respective values for FRAP, TRAP, and TEAC according to published values (58, 59). Greek tables for the composition of Greek foods were used to analyze composite foods (56). It is noted that the estimation of DAC with a similar methodology has been previously reported in the ATTICA study (63). Smoking and physical activity status were assessed as previously described (55).

### Basic biochemical measurements

Basic biochemical measurements (serum glucose, triacylglycerols, total cholesterol, and HDL cholesterol) were performed as described elsewhere (55).

### Determination of PAF levels in blood

PAF was isolated from blood, purified, and determined as previously reported (64). Whole blood with ethanol was centrifuged at 500 × g for 20 min, and the resulting supernatant (containing free PAF) and pellet (containing bound PAF) were extracted according to the Bligh–Dyer method. Subsequently, purification was followed by chromatography, and PAF levels were determined by a biological assay (64). Total PAF represents the sum of free PAF and bound PAF.

### Determination of lyso-PAF-AT, PAF-CPT, and PAF-AH activities in leukocyte homogenates and lp-PLA_2_ in serum

Leukocyte homogenate isolation is described elsewhere (18). For the determination of Lyso-PAF-AT activity, leukocyte homogenates were incubated with lyso-PAF and acetyl-CoA (18). For the determination of PAF-CPT activity, CDP-choline and 1-O-hexadecyl-2-acetyl-*sn*-glycerol were used (18). After stopping the reactions, the produced PAF was measured (64). Enzymatic activity was expressed as a specific activity in pmol/min/mg protein.

PAF-AH and Lp-PLA_2_ activities in leukocyte homogenates and serum, respectively, were determined by the trichloroacetic acid precipitation method using [^3^H] PAF as a substrate (18). The enzyme activity was expressed as nmol of PAF degraded per minute per milligram of leukocyte homogenate protein or per milliliter of serum. All assays were performed in duplicate.

### Erythrocyte fatty acid determination and estimation of desaturase indices

The erythrocyte fatty acid profile was determined by gas chromatography (Agilent HP-6890, Avondale, PA, USA) with a flame ionization detector as described elsewhere (45). Peak identification was accomplished by means of a standard mixture of 37 FAME (Sigma L9405, St Louis, MO, USA). Of the 46 fatty acids identified, 34 that comprised >0.01% of total fatty acids were considered (45).

Desaturases were indirectly estimated by fatty acid product/precursor ratios. Desaturase 5 (D5) was calculated as the ratio 20:4n6/20:3n6, D6 as the ratio 18:3n6/18:2n6, desaturase 9 (D9) C16 as the ratio 16:1n−7/16:0, and desaturase 9 (D9) C18 as the ratio 18:1n−9/18:0 (45).

### Statistical analysis

Normality was tested with the Kolmogorov–Smirnoff criterion. Normally distributed continuous variables are presented as mean ± standard deviation, while skewed variables are presented as medians and 25th−75th quartiles. Categorical variables are presented as relative frequencies (%). A *t*-test or Mann–Whitney test was applied for comparisons of parametric/log-transformed or non-parametric variables, respectively. A chi-square test was used to compare categorical variables between groups. Pearson partial correlation coefficients (after ranking variables) were evaluated to test correlations after adjustments for age, body mass index (BMI), and sex (if applicable). Given the strong differentiation between men and women in PAF metabolism (45) and PA (65), sex-specific analysis was also performed.

All reported *p*-values were two-sided (significance level 5%). The SPSS v22 software was used for statistical analysis (IBM Corp., Released 2013, Armonk, NY).

## Results

### Basic characteristics of participants

The basic clinical, anthropometric, and biochemical characteristics of the volunteers are shown in Table 1. Men had a higher waist circumference and a higher lean mass compared to women. Women had higher total body fat (%) and leg fat, as well as lower R and PA compared to men. The levels of PAF and the specific activities of its metabolic enzymes are shown in Table 2. In this group, PAF levels and its biosynthetic enzymes did not differ between sexes. The specific activities of both catabolic enzymes, namely, Lp-PLA_2_ and leukocyte PAF-AH, were higher in men.

**Table 1:** Basic characteristics of participants.

	**Total (*****n*** = **39)**		**Men (*****n*** = **20)**		**Women (*****n*** = **19)**		
	**Mean or median**	**SD or 25th**−**75th**	**Mean or median**	**SD or 25th**−**75th**	**Mean or median**	**SD or 25th**−**75th**	**p-value**
Age (years)	47.3	12.8	48.42	12.456	46.3	13.5	0.6
Current smokers (%)	33%		22%		44%		0.1
MET (min/week)	676	272–1,830	693	330–17,86	676	272–1,830	0.6
BMI (kg/m^2^)	27.3	5.2	28.3	4.4	26.4	5.8	0.2
Waist circumference (cm)	87.2	134.6	95.1	98.3	79.7	121.8	**0.001**
% Total body fat	33.1	8.9	28.2	5.6	37.7	9.2	**< 0.001**
Total lean mass (kg)	47.8	8.1	57.8	8.1	38.2	7.0	**< 0.001**
Arms lean mass (kg)	6.6	1.1	6.31	0.84	3.8	0.78	**< 0.001**
Leg lean mass (kg)	18.7	2.8	18.43	2.79	12.4	2.43	**< 0.001**
DXA ROI fat (kg)	3.0	1.3	3.3	0.8	2.7	1.6	0.1
Total cholesterol (mmol/L)	5.67	1.30	5.84	0.90	5.52	1.60	0.4
LDL cholesterol (mmol/L)	3.93	1.1	4.04	0.78	3.82	1.35	0.5
HDL cholesterol (mmol/L)	1.13	0.26	1.08	0.22	1.18	0.30	0.2
Triacylglycerols (mmol/L)^a^	1.33	0.72	1.54	0.63	1.13	0.75	0.07
Glucose (mmol/L)	4.9	4.8–5.4	5.1	4.8–5.5	4.9	4.6–5.4	0.4
Insulin (U/ml)^a^	14.6	2.3	14.9	2.1	14.3	2.6	0.4
PA (°)	5.79	0.72	6.15	0.59	5.44	0.66	**0.001**

Data are presented as mean ± standard deviation for normally distributed variables. Otherwise, data are presented as the median (lower-upper quartile) (25th−75th). Student's *t*-test was used to compare means for normally distributed variables. The Mann–Whitney test was used to compare means for non-normally distributed variables. The chi-square test was used to compare categorical variables between groups.

BMI, body mass index; HDL, high-density lipoprotein HOMA, homeostasis model assessment; LDL, low-density lipoprotein; MAC, mid arm circumference; MET, metabolic equivalents; PA, phase angle; ROI, region of interest.

^a^Values were log-transformed prior to comparisons to achieve normality.

Significant differences are shown in bold.

**Table 2:** PAF and its metabolic enzymes.

	**Total**		**Men**		**Women**		**p-value**
	**Mean or median**	**SD or (25th**−**75th)**	**Mean or median**	**SD or (25th**−**75th)**	**Mean or median**	**SD or (25th**−**75th)**	
Free-PAF^a^ (pmol/ml)	0.014	0.008–0.024	0.018	0.006–0.024	0.014	0.009–0.030	0.8
Bound-PAF^a^ (pmol/ml)	0.021	0.008–0.413	0.017	0.008–0.092	0.040	0.011–0.711	0.1
Total-PAF (pmol/ml)	0.045	0.029–0.511	0.042	0.030–0.333	0.051	0.028–0.731	0.4
PAF-CPT^a^ (pmol/min/mg)	732	346–1,380	789	346–1,842	720	323–1,088	0.7
Lyso-PAF-AT (pmol/min/mg)	75.3	44.0–95.1	75.325	48.1–95.1	76.9	34.3–108.3	0.7
Lp-${\text{PLA}}_{2}^{\text{a}}$ (nmol/min/ml)	22.87	6.064	25.31	5.195	20.55	6.032	**0.005**
PAF-AH (pmol/min/mg)	364	277–422	373	350–462	340	244–379	**0.009**

Data are presented as mean ± standard deviation for normally distributed variables. Otherwise, data are presented as median and lower-upper quartile (25th−75th). Student's *t*-test was used to compare means for normal values or log-transformed parameters. The Mann–Whitney test was to compare means for non-normal values.

^a^Variables were log-transformed prior to comparisons to achieve normality.

Lp-PLA_2_, lipoprotein-associated phospholipase A_2_; Lyso-PAF-AT: acetyl-CoA, lyso-PAF acetyltransferase; PAF, platelet-activating factor; PAF-AH, PAF acetylhydrolases; PAF-CPT: CDP-choline, 1-alkyl-2-acetyl-*sn*-glycerol cholinephosphotransferase.

Significant differences are shown in bold.

### Dietary intake of participants

The dietary intake of the participants is shown in Supplementary Table 1. The median energy intake was 2, 216 kcal. The median fat, protein, and carbohydrate intake was 39, 12.7, and 33.7%, respectively. The adherence to the Mediterranean diet assessed with the MedDietScore was 33.6 ± 5.9, and the estimation of DAC was 20.8 ± 6.5 mmol/day for FRAP, 8.0 ± 2.7 mmol/day for TRAP, and 7.9 ± 2.5 mmol/day for TEAC (means ± SD). No sex differences were documented. It is noted that PAF levels were inversely related to DAC, as shown in Supplementary Table 2.

### Erythrocyte fatty acid composition

The erythrocyte fatty acid composition is presented in Supplementary Table 3. The most abundant fatty acids were SFA and polyunsaturated fatty acids (PUFA), followed by MUFA with a median content of 36.2, 34.2, and 18.5%, respectively. The mean n−6 and n−3 content of erythrocytes was 26.4 and 8.0%, respectively. Mean D5 was 8.8, median D6 was 0.000, and median D9 was 0.014. Women had higher estimated activities in D5 and D9.

### Relationship of PA with PAF and activity of PAF metabolic enzymes

PAF levels were negatively correlated with PA in men (rho = −0.627, *p* = 0.01), while the activity of its catabolic enzyme Lp-PLA_2_ was positively correlated with PA in the total sample (rho = 0.651, *p* < 0.001) and women (rho = 0.780, *p* < 0.001). All the associations were adjusted for age, sex, and BMI. It is noted that the relationship between Lp-PLA_2_ and PA remained significant after further adjustment for LDL-cholesterol, which is the main carrier of LpPLA_2_ (rho = 0.450, *p* = 0.07 in the total sample, and rho = 0.613, *p* = 0.009 in women). In alternative models adjusted for ROI fat instead of BMI, the observed relationships were similar.

### Relationship of PA to anthropometric characteristics, erythrocyte fatty acid composition, and dietary intake

As far as anthropometric characteristics are concerned, PA was inversely correlated with fat indices in age/BMI/sex adjusted analysis for the total population and in particular with hip circumference (rho = −0.405, *p* = 0.01), %body fat (rho = −0.361, *p* = 0.03), central fat as expressed with a ROI area (rho = −0.463, *p* = 0.004), legs fat (rho = −0.382, *p* = 0.02), and arms fat (rho = −0.358, *p* = 0.03) (total sample).

As far as erythrocyte fatty acid content is concerned, PA was negatively associated with DHA (rho = −0.510, *p* = 0.03), total n-3 (rho = −0.488, *p* = 0.04), and the omega-3 index (rho = −0.509, *p* = 0.03) in men after adjustment for age and BMI. Moreover, D6 was negatively associated with PA in the total sample (rho = −0.404, *p* = 0.01) and both sexes (rho = −0.651, *p* = 0.005 in men and rho = −0.413, *p* = 0.09 in women).

Energy intake was positively correlated with PA (rho = 0.410, *p* = 0.02), and negatively associated with % PUFA intake (rho = −0.412, *p* = 0.01) after adjustment for BMI, age, and sex, while positive associations were documented for the DAC in women (Table 3). The MedDietScore was not associated with PA.

**Table 3:** Partial correlation coefficients between PA and selected dietary parameters.

	**Total sample (*n* = 39)**	**Men (*n* = 20)**	**Women (*n* = 19)**
	**Phase angle (**°**)**		
MedDietScore	0.172	0.065	0.282
	0.3	0.8	0.2
**DAC indices**			
FRAP	0.322	0.423	0.412
	0.06	0.1	0.1
TRAP	0.235	0.338	0.331
	0.1	0.2	0.1
TEAC	0.285	0.124	**0.513**
	0.1	0.6	**0.03**

Pearson partial correlations are shown after adjustments for age, sex, and BMI (total sample) or age and BMI (sex-specific analysis). *P*-values are shown below each correlation coefficient.

DAC, dietary antioxidant capacity; FRAP, ferric-reducing antioxidant power; TRAP, total radical-trapping antioxidant parameters; TEAC, Trolox-equivalent antioxidant capacity.

Significant correlations are shown in bold.

## Discussion

The present study documented first-time associations of the PA with PAF and its metabolic enzymes, erythrocyte fatty acid content, and DAC in healthy participants. More particularly, a gender-dependent nature was observed since PAF levels were negatively associated with PA in men, while its catabolic enzyme, Lp-PLA_2_, was positively associated with PA, especially in women. Moreover, the DAC based on FRAP had a borderline association with PA values in the whole population (*p* = 0.06), while the DAC based on TEAC showed a significant relationship with PA in women, suggesting a role of exogenous antioxidants in cellular health. Bound PAF levels, reflecting PAF that is strongly bound to cellular structures, were inversely related to FRAP, TRAP, and TEAC in the whole population.

In the present study, PA was 6.15° for men and 5.44° for women, which is lower than values recently suggested for healthy subjects (7.3° for men and 6.4° for women aged 18–48 years) (65). The PA was higher in men and was negatively associated with adiposity measures of total and localized fat, as previously reported (1, 65).

Several inflammatory markers, such as C-reactive protein, interleukin-6, and tumor necrosis factor-a (TNF-a), have been negatively associated with PA (8, 9, 66). In the present study, PAF, a potent lipid inflammatory mediator, was negatively associated with PA in men. It has been reported that pro-oxidative environmental stressors resulting in cell membrane damage lead to the production of PAF and PAF agonists (32). It should be noted that during the biosynthesis of PAF through the remodeling pathway, arachidonic acid is released and converted into a variety of eicosanoid mediators. The generation of inflammatory mediators, including PAF, metalloproteinases, etc., may further disrupt membrane integrity (67) and lead to lower PA values. PAF may also be related to membrane integrity either directly (30, 31) as one of the ether lipids that participates in membrane structure (68) or indirectly by modifying the antioxidant/inflammatory cellular milieu and/or the availability of fatty acids (20). Specifically, PAF and PAF agonists modulate the redox status by the production of ROS (38–41) and trigger the excretion of other pro-inflammatory mediators (69). PAF also mediates NLRP3-NEK7 inflammasome induction (70), which in turn results in cell death (71). Finally, PAFR stimulation activates the translocation of acid sphingomyelinase (aSMase) to the plasma membrane, resulting in alterations in membrane integrity and fluidity (33–35). In the same context, Lp-PLA_2_ was positively associated with PA in the whole population and in women. Lp-PLA_2_ hydrolyzes oxidatively modified phospholipids and other pro-inflammatory molecules, such as PAF (20), and it is inactivated upon LDL oxidation (37). Thus, a potentially oxidative environment, which relates to low PA, could be combined with low Lp-PLA_2_ activity. The sex-dependent nature of the observed associations of PAF metabolism with PA may be partially attributed to the already reported different PAF metabolic patterns in both sexes (72), while the role of fat distribution and related dietary habits cannot be excluded (73). It is also noted that in a pathological context, such as cancer, PA and Lp-PLA_2_ are differentially affected: PA is reduced in cases of high inflammatory burden, such as cancer and sarcopenia (49, 74), while Lp-PLA_2_ is increased (75). In addition, in a recent meta-analysis, the values of PA were associated with cardiovascular diseases (76), and Lp-PLA_2_ has been proposed as a marker (77). This observation is in line with the negative association between PA and Lp-PLA_2_ in the present study.

The data regarding the relationship of PA with fatty acids are limited, and there is no study investigating the relationship between desaturases and PA. N-3 PUFA has been positively associated with PA in patients (13, 14) and increases membrane fluidity (78). However, EPA supplements did not change PA in cancer patients (79). In our study, n-3 and the omega-3 index were negatively associated with PA, but when smoking status (rho = −0.501, *p* = 0.057 and rho = −0.476, *p* = 0.07, respectively) or circulating thiobarbituric acid reactive substances (TBARS) were considered (rho = −0.338, *p* = 0.2 and rho = −0.342, *p* = 0.1, respectively), the correlations were no longer significant, suggesting an effect of oxidative stress. It is possible that phospholipids with polyunsaturated fatty acyl residues, such as n−3, may be more easily oxidized (80). Such oxidatively truncated phospholipids can serve as mediators of TNF-a-induced cell death (80) and may damage mitochondrial integrity (81). Thus, in cases of high oxidative stress, a high erythrocyte PUFA content may disrupt cell integrity (81). The association of PA with estimated desaturase activity has not been previously assessed. In our study, D6 was negatively associated with PA, which is in line with the role of D6 in cardiometabolic diseases (82, 83). The recently reported positive associations of PAF biosynthetic enzymes with D6 may also partially explain the observed finding (45).

The relationship of diet with PA has not been thoroughly investigated. Pilot results have shown a positive association of PA with Mediterranean diet adherence (11). In the present study, macronutrient intake was not related to PA similarly to others (84), while only the DAC was positively associated with PA, suggesting a protective role of antioxidants in cell integrity. Indeed, PA has been negatively related to inflammatory and oxidative stress markers in health and disease (8, 9). Moreover, a significant positive association between PA and plasma antioxidant capacity has been reported (9), which corroborates our findings.

Limitations of our study include its cross-sectional nature, which cannot prove causality. Pitfalls in dietary assessment may exist since subjects may not have appropriately estimated their intake. For this reason, multiple dietary recalls were used, along with the FFQ and erythrocyte PUFA. As far as the DAC is concerned, published values on raw Italian foods were used (58, 59), which may differ from the actual values of Greek foods or the *in vivo* activity of cooked foods. It is also noted that PAF has a short half-life, and its fluctuations at the time of measurement may not reveal fully its biological correlates. In the present study, its metabolic enzymes were also measured to serve as an “index” of the circulating PAF trend. Finally, a relatively small number of apparently healthy volunteers had available data on BIA and was included, since the present study was a sub-analysis of a previous study investigating PAF and its enzymes in 106 participants (55). Indeed, in a power analysis using the G-Power software (version 3.1.9.7, Kiel University), the achieved power was high for significant associations with relatively high correlation coefficients (rho = 0.4–0.6) (>90%) (such as the association of PA with DAC in woman), but low for non-significant associations with lower correlation coefficients (rho = 0.2) (~40%) (such as the association of PA with DAC in the total sample), meaning that the present study may be underpowered for several associations. The healthy status of the volunteers also implies that the observed relationships may be different in pathological conditions.

## Conclusion

PA is inversely associated with PAF levels and positively associated with Lp-PLA_2_ activity in a gender-dependent manner, indicating the involvement of PAF in the impairment of cell membrane. Moreover, PA was positively associated with high dietary antioxidant intake in apparently healthy participants. The associations of PAF and diet with PA can be explained by the fact that PAF may impair cell membranes, while antioxidants inhibit PAF generation and have a beneficial effect on cell integrity. The fact that those associations were observed in the context of an apparently healthy population emphasized the role of PA as a novel, sensitive, non-invasive, and easily determined index of PAF-mediated subclinical inflammation.

## Data availability statement

The raw data supporting the conclusions of this article will be made available by the authors, without undue reservation upon request.

## Ethics statement

The studies involving humans were approved by Bioethics Committee of Harokopio University. The studies were conducted in accordance with the local legislation and institutional requirements. The participants provided their written informed consent to participate in this study.

## Author contributions

PD and SA: conceptualization. PD: data curation, formal analysis, and roles/writing—original draft. SA: funding acquisition, resources, and supervision. PD, EF, and TN: investigation. EF, TN, and SA: methodology and writing—review and editing. PD, EF, TN, and SA: project administration. All authors contributed to the article and approved the submitted version.
