# Supplementary material for: Associations of phase angle with platelet-activating factor metabolism and related dietary factors in healthy volunteers
Source: Front Nutr. 2023 Nov 3;10:1237086. doi: 10.3389/fnut.2023.1237086 (PMC10655008; doi:10.3389/fnut.2023.1237086)
Supplement: Supplementary file 1 [file Table_1.DOCX]

Supplementary Material

Associations of phase angle with platelet-activating factor metabolism and related dietary factors, in healthy volunteers

**Paraskevi Detopoulou, Elizabeth Fragopoulou, Tzortzis Nomikos, Smaragdi Antonopoulou**** Corresponding Author: antonop@hua.gr

# Supplementary Tables

**Supplementary Table 1: Dietary intake of the participants.**

|  | **Total** |  | **Men** |  | **Women** |  | **p** |
| --- | --- | --- | --- | --- | --- | --- | --- |
|  | **Mean**  **or median** | **SD or (25th-75th)** | **Mean**  **or median** | **SD or (25th-75th)** | **Mean**  **or median** | **SD or (25th-75th)** |  |
| **Energy (Kcal) ^a^** | 2216 | 1828- 2722 | 2135 | 1787-2702 | 2262 | 1810-2857 | 0.3 |
| **Fat (% energy) ^a^** | 39 | 36-43 | 40 | 37-44 | 38 | 34-43 | 0.7 |
| **Monounsaturated fat (% energy) ^a^** | 22 | 20-26 | 23 | 20- 27 | 20 | 16-25 | 0.2 |
| **Saturated fat (% energy)** | 13.7 | 25.5 | 13.6 | 2.2 | 13.8 | 2.8 | 0.3 |
| **Polyunsaturated fat (% energy)** | 35.8 | 0.8 | 33 | 6.7 | 37.5 | 9.1 | 0.4 |
| **Protein (% energy)** | 12.7 | 21.3 | 12.7 | 2.3 | 12.7 | 1.9 | 0.8 |
| **Carbohydrate (% energy)** | 33.7 | 49.6 | 33.65 | 5.4 | 33.7 | 4.5 | 0.3 |
| **Cholesterol (mg) ^a^** | 133 | 66-214 | 111 | 65- 220 | 135 | 72-214 | 0.8 |
| **Dietary fiber (g)** | 34.0 | 15.7 | 31.4 | 12.9 | 36.5 | 18.0 | 0.3 |
| **MedDietScore (0-55)** | 33.6 | 5.9 | 33.83 | 4.7 | 33.5 | 7.1 | 0.1 |
| **DAC FRAP**  **(mmol/day)** | 20.8 | 6.5 | 19.7 | 7.2 | 21.8 | 5.7 | 0.3 |
| **DAC TRAP**  **(mmol/day)** | 8.0 | 2.7 | 7.5 | 2.8 | 8.5 | 2.5 | 0.4 |
| **DAC TEAC**  **(mmol/day)** | 7.9 | 2.5 | 7.3 | 2.2 | 8.5 | 2.7 | 0.3 |

Data are presented as mean ± standard deviation for normally distributed variables. Otherwise, data are presented as median and lower- upper quartile (25^th^- 75^th^). Student t-test was used to compare means for normal values or log-transformed parameters. Mann-Whitney test was to compare means for non- normal values.

^a^ Variables were log-transformed prior to comparisons to achieve normality.

**Supplementary Table 2: Partial correlation coefficients between PAF and DAC indices.**

|  | **Free-PAF** | **Bound-PAF** | **Total-PAF** | **PAF-CPT** | **Lyso-PAF-AT** | **PAF-AH** | **Lp-PLA_2_** |
| --- | --- | --- | --- | --- | --- | --- | --- |
| **FRAP** | **0.350**  **0.049** | **-0.426**  **0.012** | **-0.365**  **0.040** | -0.174  0.326 | -0.137  0.439 | 0.032  0.859 | 0.176  0.320 |
| **TRAP** | **0.406**  **0.021** | **-0.393**  **0.022** | **-0.353**  **0.048** | -0.093  0.601 | -0.063  0.725 | 0.002  0.990 | 0.072  0.685 |
| **TEAC** | 0.323  0.072 | **-0.491**  **0.003** | -0.303  0.092 | -0.055  0.757 | -0.072  0.687 | 0.090  0.620 | 0.139  0.433 |

Pearson partial correlations are shown after adjustments for age, sex and BMI. P values are shown below each correlation coefficient. Variables were ranked prior to comparisons to perform analysis.

DAC: Dietary antioxidant capacity; FRAP: ferric-reducing antioxidant power; Lp-PLA_2_: lipoprotein-associated phospholipase A_2_; Lyso-PAF-AT: acetyl-CoA: lyso-PAF acetyltransferase; PAF: platelet-activating factor; PAF-AH: PAF acetylhydrolases; PAF-CPT: CDP-choline: 1-alkyl-2-acetyl-*sn*-glycerol cholinephosphotransferase; TRAP: total radical-trapping antioxidant parameters; TEAC: Trolox-equivalent antioxidant capacity

**Supplementary Table 3: Erythrocyte fatty acids of the participants (% of total fatty acids).**

|  | **Total** |  | **men** |  | **women** |  | **p** |
| --- | --- | --- | --- | --- | --- | --- | --- |
| **% of total fatty acids** | **Mean or median** | **SD or (25th-75th)** | **Mean or median** | **SD or (25th-75th)** | **Mean or median** | **SD or (25th-75th)** |  |
| **14:0** | 0.20 | 0.08 | 0.20 | 0.08 | 0.19 | 0.09 | 0.7 |
| **15:0** | 0.12 | 0.06-0.66 | 0.13 | 0.10-0.66 | 0.11 | 0.00-0.68 | 0.2 |
| **16:0** | 17.3 | 15.8- 17.8 | 17.4 | 17.3-18.1 | 16.4 | 14.9-17.8 | 0.06 |
| **16:1 ω7** | 0.25 | 0.17-0.31 | 0.24 | 0.19-0.28 | 0.28 | 0.14-0.37 | 0.8 |
| **17:0^a^** | 0.29 | 0.26-0.32 | 0.30 | 0.28-0.32 | 0.28 | 0.22-0.32 | 0.6 |
| **17:1** | 0.53 | 0.43 | 0.51 | 0.45 | 0.56 | 0.42 | 0.6 |
| **18:0** | 14.2 | 12.8-14.8 | 14.7 | 14.1-15.0 | 14.0 | 12.4-14.7 | **0.009** |
| **18:1ω9** | 13.1 | 11.9-15.1 | 13.5 | 13.0-16.5 | 12.5 | 11.1- 13.5 | **0.02** |
| **18:1 ω7** | 0.84 | 0.41-0.94 | 0.84 | 0.00-1.01 | 0.84 | 0.59-0.89 | 0.6 |
| **18:2 ω6 (LA)** | 8.49 | 7.81-9.97 | 8.3 | 7.8-8.9 | 8.8 | 7.4-10.4 | 0.5 |
| **20:0** | 0.34 | 0.06 | 0.33 | 0.05 | 0.34 | 0.07 | 0.4 |
| **20:1 ω9** | 0.30 | 0.10 | 0.33 | 0.88 | 0.28 | 0.11 | 0.1 |
| **20:3 ω9** | 0.04 | 0.00-0.16 | 0.07 | 0.00-0.14 | 0.00 | 0.00-0.30 | 0.5 |
| **20:3ω6** | 1.36 | 0.31 | 1.48 | 0.32 | 1.24 | 0.25 | **0.01** |
| **20:4ω6 (AA)** | 11.0 | 10.3-12.6 | 11.4 | 10.4-12.6 | 11.0 | 10.1-12.8 | 0.5 |
| **22:0** | 0.17 | 0.00-0.45 | 0.15 | 0.00-0.23 | 0.32 | 0.00-0.60 | 0.09 |
| **22:1 ω9** | 0.02 | 0.00-0.15 | 0.00 | 0.00-0.10 | 0.04 | 0.00-0.85 | 0.3 |
| **22:5ω6** | 0.40 | 0.31-0.52 | 0.44 | 0.34-0.49 | 0.36 | 0.29-0.66 | 0.3 |
| **24:1ω9** | 3.00 | 1.07 | 3.11 | 1.10 | 2.88 | 1.05 | 0.5 |
| **20:5ω3 (EPA)** | 0.42 | 0.32-0.70 | 0.43 | 0.34-0.68 | 0.39 | 0.20-0.77 | 0.7 |
| **22:6ω3 (DHA)** | 5.29 | 1.19 | 5.31 | 0.99 | 5.2 | 1.39 | 0.9 |
| **22:4 ω6** | 2.46 | 0.75 | 2.56 | 0.81 | 2.36 | 0.69 | 0.4 |
| **22:5 ω6** | 0.40 | 0.19 | 0.42 | 1.51 | 0.38 | 0.22 | 0.5 |
| **22:5 ω3 (DPA)** | 2.12 | 0.42 | 2.10 | 0.34 | 2.15 | 0.50 | 0.9 |
| **22:6 ω6** | 0.69 | 0.42-0.97 | 0.66 | 0.41-1.11 | 0.76 | 0.30-0.97 | 0.7 |
| **24:0** | 2.59 | 0.99 | 2.57 | 0.65 | 2.61 | 1.27 | 0.9 |
| **24:1 ω9** | 3.0 | 1.07 | 3.11 | 1.10 | 2.88 | 1.05 | 0.5 |
| **SFA** | 36.2 | 32.1-37.2 | 36.5 | 35.4-37.2 | 33.3 | 31.5-37.1 | 0.06 |
| **MUFA** | 18.5 | 16.7-21.5 | 18.6 | 17.3-22.7 | 18.3 | 16.4-20.0 | 0.4 |
| **PUFA** | 34.2 | 32.8-35.4 | 34.1 | 33.1-35.4 | 34.3 | 32.8-35.4 | 0.9 |
| **n-6** | 26.4 | 3.25 | 26.3 | 2.27 | 26.6 | 4.06 | 0.8 |
| **n-3** | 8.0 | 1.66 | 7.95 | 1.34 | 8.0 | 1.96 | 0.8 |
| **Omega-3 index** | 5.81 | 1.44 | 5.82 | 1.17 | 5.8 | 1.70 | 0.9 |
| **D-5** | 8.80 | 1.95 | 8.04 | 1.78 | 9.55 | 1.86 | **0.01** |
| **D-6** | 0.0000 | 0.0000-0.0298 | 0.0000 | 0.0000-0.0000 | 0.009 | 0.000-0.056 | **0.007** |
| **D-9** | 0.014 | 0.010-0.017 | 0.012 | 0.011-0.016 | 0.016 | 0.008-0.020 | 0.4 |

Data are presented as mean ± standard deviation for normally distributed variables. Otherwise, data are presented as median and lower- upper quartile (25^th^- 75^th^). Student t-test was used to compare means for normal values or log-transformed parameters. Mann-Whitney test was to compare means for non- normal values.

^a^ Variables were log-transformed prior to comparisons to achieve normality.
